# Role of Mobile Phone Use in Spreading Multi-Drug Resistant Bacteria Implicated in Causation of Diarrheal and Nosocomial Infections at Kitale County Hospital

**DOI:** 10.24248/eahrj.v7i2.736

**Published:** 2023-11-30

**Authors:** Jumba Sande Godfrey, Kevin Mbogo, Antony W. Wekesa

**Affiliations:** a Kitale County Hospital, Laboratory department, Trans Nzoia County; b Jomo Kenyatta University of Agriculture and Technology, Biochemistry department, Juja, Kenya; c Division of Vector Borne and Neglected Tropical Diseases, Bungoma County

## Abstract

**Background:**

Rapid advancement and penetration of mobile phone technology has made the devices indispensable professional, social, and networking tools. However, the impact of their use in the spread of multi-drug resistant diarrheal-causing bacteria is less understood.

**Objective:**

The aim of this study was to determine the practices of mobile phone use among healthcare workers, paediatric patients' caretakers, and paediatric inpatients with diarrhoea at Kitale County Referral Hospital, and identify the associated risk of spread of bacteria, including multi-drug resistant strains on the mobile phones.

**Method:**

Questionnaires were administered to research participants and swabs were collected from mobile phones of consenting healthcare workers and paediatric patients' caretakers for further analysis. Stool samples were also collected from paediatric study participants diagnosed with gastroenteritis. Culture was done following standard microbiological procedures. Isolate identification, antibiotic susceptibility profile, and MDR phenotypes were tested using the Vitek 2 Compact microbiology analyzer. Gram-negative MDR isolates were then screened for selected carbapenemase genes using multiplex real-time PCR.

**Results:**

Only 38% of healthcare workers sanitize their handsets during or after work. The most common mobile phone bacterial isolate was Enterococcus faecalis (28.95%) followed by Staphylococcus aureus (18.42%). 58% of stool sample isolates were Vibrio cholera 01 serotype followed by Escherichia coli 0157.H7 (20%). Methicillin-resistant Staphylococcus aureus (43%) and Vancomycin Resistant Enterococcus (6%). BlaVIM was the most commonly detected gene from five isolates, including Vibrio cholera 01

**Conclusion:**

The most common pathogen circulating on the mobile phones of healthcare workers and patients' caretakers at Kitale County Hospital is Enterococcus faecalis.

## BACKGROUND

Rapid advancement in mobile phone technology has led to the devices becoming indispensable social, professional and networking tools however, their potential for infection spread has not been fully explored.^1^ The immense benefits of mobile phone use often mask its potential infectious hazards to health. Constant handling and sharing of mobile phone devices by different users in the healthcare environment, poor hand hygiene practices, and heat generated by them create a conducive environment for the multiplication of all types of micro-organisms normally found on the skin as well as bacteria associated with nosocomial infections contributing significantly to the spread of healthcare-associated infections.^2^ Concerns on safety and subsequent legislation to restrict use have largely been based on interference with electronic and other equipment but less policy attention has been paid to infectious risk.^3^ A study done at Khaja Banda Nawaz Teaching and General hospital in India revealed that 94.05% of swabs from mobile phones of healthcare workers and non-health care workers had microbes, predominantly (80.14%) coagulase-negative staphylococci (CONS) followed by gram-positive bacilli (68.1%), Methicillin Sensitive *Staphylococcus aureus*-MSSA (19.8%), Methicillin Resistant *Staphylococcus aureus*-MRSA (17%), *Pseudomonas spp* (9.9%), *Micrococcus spp* (9.9%), *Enterococcus spp* (7.1%) and E. coli (4.9%).^4^ With increased antibiotic resistance traits among these species and the fact that 92.4% of inpatients consider the use of mobile phones as vital, there is a potential of mobile phones serve as agents of infection transmission within the hospital.^5^

The infectious dose varies greatly from as many as 10^8^ for enterotoxigenic *Escherichia coli* (ETEC) to as few as 10 organisms for enterohemorrhagic *E. coli 0.157. H7* strain. *Salmonella typhi* the causative organism for enteric fever has been proven to survive on fingertips for up to eighty minutes while *Escherichia coli* can survive on inanimate surfaces outside the host for between 1.5 hours to 16 months. There is however no local data from Kenya in support of the same.^6–8^

A hospital-based study was carried out to determine mobile phone use behaviour, the diversity of bacterial isolates from mobile phones of health care workers, paediatric patients' caretakers, stool samples from paediatric inpatients seeking services at Kitale County Referral Hospital, their antibiotic sensitivity profile, Multi Drug Resistance (MDR) phenotypes, and Carbapenem resistance genotypes. The aim of the study was to decipher the role of mobile phone use in the spread of diarrheal, nosocomial and multidrug-resistant organisms in the hospital.

## MATERIALS AND METHOD

### Study Design, Site, and Population

A hospital based descriptive study was carried out from March 2016 to April 2017 at Kitale County Referral Hospital, a 250-bed capacity hospital that admits an average of nine paediatric patients daily, 273 paediatric patients monthly, and 3,276 paediatric patients annually. The hospital is located at: (latitude −1.0164317 Longitude −35.00890020593023 and Elevation-1906 m) in Kitale town of Trans Nzoia County. The County has an area of 2,492.5 km^2^ and a population of 990341 persons.^9^

### Inclusion Criteria

Study participants included; consenting healthcare workers who had a mobile phone, and had been working in their current stations for at least one week; paediatric patients aged between 1 and 12 years, diagnosed with gastroenteritis in the last 72 hours and paediatric parents or guardians who owned a mobile phone and had been nursing their sick child throughout their inpatient stay period.

### Sample Size Determination and Sampling

With the assumption that 50% of healthcare workers and caretakers use mobile phones without washing their hands during and after attending paediatric patients, and given the 95% confidence interval and acceptable margin of error of 5%, the Cochran's 1977 formula was used to yield a sample size of 384.^10^ Since the target population was small (less than 10,000), Cochran's correction formula was used to calculate the final sample size of 365 subjects.^11^

Probability proportional to size sampling method was used to determine number of each of the study participants to be enrolled. The working sample size was 423 participants.^12^

### Collection and Analysis of Swabs from Mobile Phones

Swabs from mobile phones of healthcare workers and paediatric patients' caretakers were collected and immediately placed in individual bottles of sterile brain heart infusion broth (Himedia, Lot-257398, 2016) prepared according to manufacturer instructions. They were then labelled with participants' unique study numbers, and incubated aerobically overnight at 37°C. A subculture was subsequently done from the broth culture to sheep blood agar (SBA base - Himedia, Lot-316699, 2017), Chocolate blood agar (CBA base-Himedia, Lot-316699, 2017) and MacConkey agar plates (Bd-Lot-7076631, 2017), and incubated aerobically overnight at 370C. A well-isolated colony of the bacteria obtained was then loaded into the Vitek 2 compact analyser (Biomerieux, software 9.2.0), following manufacturer instructions, using selected identification cards (Biomerieux, GN, 2017 and GP, 2017) and sensitivity cards (Biomerieux, ASTGN83, 2017 and ASTP580, 2017). Organism identification was accepted when the confidence level was ≥ 85% probability. Gram-stained smears from the pure cultures were prepared and examined microscopically as a quality control measure to confirm the purity of the colonies.

### Collection and Analysis of Stool Samples

Stool samples were collected from paediatric patients with diarrhoea; they were labelled appropriately and inoculated into Sorbitol MacConkey agar (Himedia, Lot-298902, 2017), alkaline peptone water (APW-Himedia, Lot-208197, 2014) and Selenite F broth (SF-Himedia, Lot-292817, 2017) within one hour of collection. Culture and identification was done as per established standard procedures.^12^

Identified species were then loaded into the Vitek 2 compact analyser (Biomerieux, software 9.2.0) for antimicrobial susceptibility testing. Gram-negative isolates with detected antibiotic resistance phenotypes were archived for molecular testing.

### Deoxyribonucleic Acid (DNA) Extraction

DNA extraction from bacteria was done using the QIAcube machine (Qiagen, software version FIM-50-002_PLC_MP.prs, 2014) and QIAamp® DNA mini QIAcube extraction kit. The organisms were first sub cultured in sterile Tryptic soy broth (Oxoid, Lot-2401787, 2018), a pellet from the broth culture prepared and loaded into the QIAcube, following manufacturer instructions. An extraction control and internal control were included in the automated process to monitor for any contamination.^13^

### Amplification of Antibiotic Resistance Genes

Screening of Carbapenem drugs resistance genes (blaKPC), blaOXA-48, blaVIM, blaNDM, and blaIMP) was done using multiplex real time PCR. PCR reaction mix was prepared following the manufacturer instructions for Sacace (Italy, lot: 21J15M001, 27H15J301) commercial kits, and PCR tubes loaded into Rotor Gene Q thermocycler (Qiagen, software version 2.1.0.9, 2015) following the manufacturer's instructions. Positive and negative controls provided were also included in the run. Fluorescent signal was detected in the FAM (blaVIM and blaKPC), HEX (blaIMP and blaOXA-48-similar), and CY5 (blaNDM) Channels. Significant fluorescence detection as per the kit instruction manual was when the acceptable cycle threshold (Ct) for the positive control was <30 and <38 for positive samples, zero Ct value for the negative control, extraction control, and no template control. Results were accepted as significant only when all the controls above passed correctly.

### Data Analysis

Data in the study questionnaires was entered into a Statistical Package for Social Sciences (SPSS-Version 20) database and analysed. Descriptive statistical variables as well as variables on mobile phone use perceptions and behaviour were established from study participants' responses. laboratory-generated reports on antimicrobial susceptibility profiles were entered and analysed using WHONET 2017, a software created by the world health organization specifically for analysis of microbiology laboratory data with focus on antimicrobial resistance surveillance.^14^

### Ethical Consideration

Ethical clearance for the study was sought from the Kenyatta University Ethical Review Committee (KU/R/COMM/51/604). Research authorization was also sought from the national commission for science technology and innovation (NACOSTI/P/16/47112/9003) and Kitale County Referral Hospital Research Committee. Written informed consent and/or assent was obtained from each study participant or their parents/guardians.

## RESULTS

A total of 424 participants were recruited into the study, out of which 187 were paediatric patients with diarrhoea, 187 patient's caretakers, and 50 healthcare workers distributed across all cadres. The age range for health care workers was 21 to 58 years with a mean age of 28 years, paediatric participants age ranged from 1 to 12years with the mean age of 4 years. Paediatric participants' caretakers' age ranged from 15 to 70 years with the average being 30 years. The majority of healthcare workers had worked in their current stations for over a year (52%) while a minority had been stationed there for over 1 week.

### Health Care Workers' Mobile Phone Use Bio-risk Assessment

Fifty-eight percent of the health care workers were female while 38% male. Majority of the participants (46%) were laboratory personnel while from the clinical team, the majority being clinical officer interns and staff (22%). Most health care workers (68%) admitted to receiving calls while attending to patients with 70% consenting to sharing their mobile phones with patients or their caretakers when requested.

Despite the majority of health care workers (80%) being aware that mobile phones can be fomites for harmful bacteria, only 38% had institutionalized the habit of sanitizing their handsets with 70% alcohol solution during or after work. Sixty-six percent (66%) of healthcare workers opposed the idea of restriction of mobile phone use within the hospital set up.

### Patients Caretakers' Mobile Phone Use Bio-risk Assessment

Majority of patients' caretakers (37.6%) had owned their mobile phones for between 1–2 years and 29% admitted to sharing the handsets and/or the accessories with other caretakers while in hospital. Majority (84.4%) admitted to receiving calls while changing baby diapers, (94.2%) while cooking back at home and (94.6%) while feeding their children. Majority (71.5%) were unaware that mobile phones could be carrying harmful bacteria and 95.2% of caretakers allowed their children to play with their handsets within the hospital and back at home.

### Bacterial Isolates From Mobile Phones and Stool Samples

**FIGURE 1:**
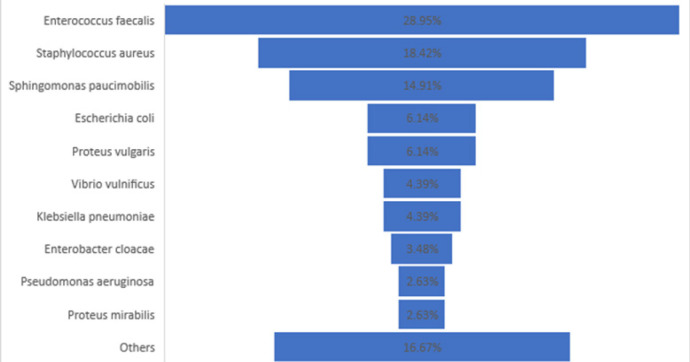
Chart Illustrating the Percentage Distribution of Isolates from Mobile Phones of Health Care Workers and Patients' Caretakers at Kitale County Hospital

**FIGURE 2:**
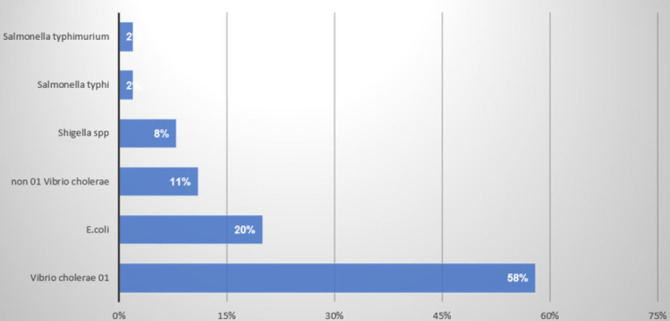
Percentage Illustration of Isolates Recovered from Stool Samples of Paediatric Patients with Diarrhoea at Kitale County Hospital

The most common bacterial isolate from mobile phones was *Enterococcus faecalis* (28.95%) followed by *Staphylococcus aureus* (18.42%), *Sphingomonas paucimobilis* (14.91%), and *Escherichia coli* at 6.14%. Fifty-eight percent of stool sample isolates were *Vibrio cholera 01* serotype followed by *Escherichia coli 0157.H7* (20%) and *Shigella* species (11%). (Figure 1, 2)

### Antimicrobial Susceptibility Profile of Stool and Mobile Phone Isolates

Among the isolates from stool samples, the highest resistance was to Trimethoprim/Sulphamethoxazole drug (87%) followed closely by Cefotaxime (86%), Cefepime (86%), and Ampicillin (83%). The least antibiotic resistance was to Gentamicin (17%), Amikacin (15%), and Levofloxacin (9%).

**FIGURE 3:**
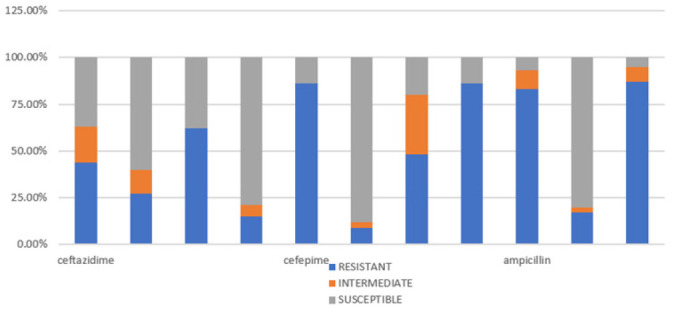
Percentage Illustration of Antimicrobial Susceptibility Profile of Pathogenic Isolates from Stool Samples (N=82).

**FIGURE 4:**
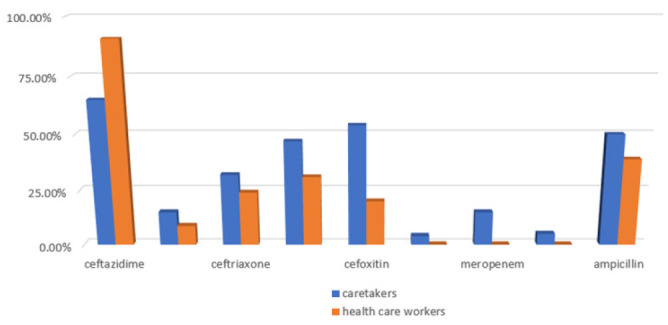
Comparison of Antibiotic Resistance Profile between Isolates from Mobile Phones of paediatric Patients' Caretakers and Health Care Workers.

Most of the isolates from mobile phones of paediatric patients' caretakers showed higher resistance profiles than those of healthcare workers except for the Ceftazidime antibiotic where isolates from health professionals exhibited higher resistance (91%) compared to 65% resistance to the same drug by isolates from mobile phones of caretakers. Resistance to Levofloxacin, Meropenem, and Vancomycin was however low in both categories. Forty-three percent of *Staphylococcus aureus* isolates were Methicillin-resistant *Staphylococcus aureus* (MRSA) while 57% Methicillin sensitive *Staphylococcus aureus* (MSSA). Six percent of the *Enterococcus* species were Vancomycin-Resistant *Enterococcus* (VSE) while 96% were Vancomycin Sensitive *Enterococcus* (VSE). In the larger Enterobacteriaceae family together with other gram-negative non-spore-forming rods, 33.63% were Extended spectrum beta Lactamases (ESBL) producers while 14.36% were Carbapenem-Resistant Enterobacteriaceae-CRE. (Figure 3, 4)

### Molecular Screening of Expression of Carbapenem Resistance Genes

The carbapenem resistance genetic determinant blaVIM was the most commonly detected carbapenemase gene from five different isolates, followed by blaOXA 48 SIMILAR from four isolates and blaNDM in that order. Carbapenemase resistance genes blaKPC and blaIMP were not detected in isolates analysed in this study (Table 1)

**TABLE 1:** Isolates, Cycle Threshold (Ct) Values, and Detected Carbapenemase Gene Type

Isolate	BlaNDM Ct value	BlaVIM Ct value	BlaIMP Ct value	BlaOXA-48 Ct value	BlaKPC Ct value	GENES DETECTED
*Escherichia coli*	32.55					BlaNDM
*Escherichia coli*		30.66		28.39		BlaVIM
*Escherichia coli*						BlaOXA48
*Vibrio cholera*		31.97				BlaVIM
*Vibrio cholera*		31.08				BlaVIM
*Enterobacter cloacae spp cloacae*	15.09					BlaNDM
*Enterobacter cloacae spp cloacae*		30.85				BlaVIM
*citrobacter amalonaticus*		32.10				BlaVIM
*Sphingomonas paucimobillis*				35.90		blaOXA-48-Similar
*Proteus hauseri*				27.19		blaOXA-48-Similar
*Enterobacter aerogenes*				33.65		blaOXA-48-Similar

The acceptable cycle threshold (Ct) is, <38 for positive samples.

**FIGURE 5:**
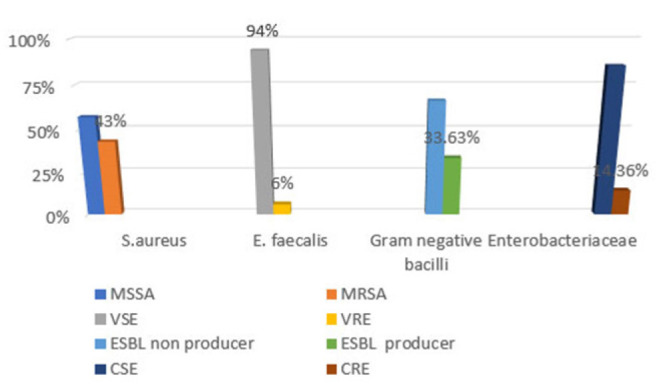
Percentage Illustration of Isolates with Multi Drug Resistant Phenotypes

## DISCUSSION

Healthcare professionals in their routine patient care and patients' caretakers constantly handle mobile phones in clinical setups for communication, reference to clinical notes, medical dictionaries, journals, and drug indexes, with minimal or no disinfection practices. This norm makes patients vulnerable to cross-infection as well as nosocomial infections since it happens in their vicinity.^5,15,16^ This study was able to demonstrate the presence of carbapenem-resistant gram-negative isolates as well as other multi drug resistant isolates from mobile phones and stool samples of study participants as well as other multi drug resistant organisms.

The predominant pathogenic isolate from mobile phones was *Enterococcus faecalis* (28.95%), followed by *Staphylococcus aureus* (18.42%), *Sphingomonas paucimobilis* (14.91%), *Escherichia coli* (6.14%) and Proteus vulgaris (6.14%). Similar organisms have been isolated in other studies.^1,17,18^ There was, however, variation in the predominant isolate among the comparative studies. For example, in the study done among health care workers, students, and other diverse groups of professionals in Nigeria, the predominant pathogenic isolate was *Staphylococcus aureus* (30.6%), while in Nepal, *Bacilli species* (20.4%) predominated. In an Indian study done on mobile phones of healthcare workers and non-healthcare workers in a hospital environment, *Acinetobacter baumanii* was the most common isolate.^1,17,18^

Bacterial isolates from mobile phones of paediatric patients' caretakers showed higher resistance profiles than those of healthcare workers except for Ceftazidime where isolates from health professionals exhibited higher resistance (91%) compared to 65% resistance to the same drug by isolates from mobile phones of caretakers. The results contrast those in Bangladesh where analysis of isolates done in from mobile phones of healthcare workers from different hospitals revealed 45.3% resistance to Ceftazidime.^16^

The isolates recovered from paediatric stool samples depicted the highest resistance to Trimethoprim/Sulphamethoxazole drug (87%) followed closely by Cefotaxime (86%), Cefepime (86%), and Ampicillin (83%). Comparable results for Ampicillin (81%) and Trimethoprim / Sulphamethoxazole (67-100%) have been found, in studies conducted on children from Nyanza province, western Kenya.^19,20^

The study coincided with a cholera outbreak in 2017 and 31 vibrio cholera 01 isolates were recovered from stool samples. They were 100% susceptible to Meropenem, Levofloxacin, and Gentamicin. In comparison to a study carried out in Kisumu county western Kenya where 101 vibrio cholerae isolates were recovered from patients during the 2017 epidemic, the susceptibility profile of the isolates to gentamycin was 100% despite variation in the choice of susceptibility testing methods and a larger number of isolates tested. This suggests the possibility of a similar *Vibrio cholera* strain affecting the residents of the two neighbouring counties located in the same region.^4^

Previous studies have highlighted mobile phones as potential reservoirs of multi-drug resistant bacterial isolates. These findings are well corroborated by our study where Methicillin-resistant Staphylococcus aureus (MRSA), Vancomycin-Resistant Enterococcus (VRE), ESBL producing gram-negative bacteria and carbapenemase resistant Enterobacteriaceae were isolated from both mobile phones and stool samples. Noted differences in the prevalence, could be attributed to the variations in the prevalence of organisms associated with nosocomial infections in the different research settings.^21,22^

Beta-Lactamases are classified based on various schemes with the most useful describing both the structural and functional profile. Carbapenemases are the most versatile *β*-lactamases with the ability to hydrolyze not only the carbapenems but also other beta-lactam antibiotics.^23,24^ Carbapenem drugs have been the drug of choice for infections caused by multi-drug resistant gram-negative organisms and the emergence of resistance to these drugs of last resort, is a serious public health concern threatening the existence of the human race.^23–25^

The most common Carbapenemase gene was blaVIM isolated from four different isolates namely *Enterobacter cloacae, Escherichia coli, Citrobacter Amalonaticus, and Vibrio cholera.* In other studies, the gene has been detected in *Citrobacter Freundii*, *Enterobacter cloacae,* and *Escherichia coli*.^24,26^

The detection of blaVIM from two different *Vibrio cholera 01* isolates is a significant finding and a public health concern due to the epidemic nature of the organism. The carbapenemase gene has been documented only in non-01/non-0139 avian isolates and not in Vibrio cholera 01 strains.^27^

OXA-Carbapenemases (OXA-48 and OXA-162) denoted as blaOXA similar were originally isolated in turkey but currently isolates with these genes are among the most commonly isolated bacteria in the family Enterobacteriaceae. The genes were detected in *Escherichia. coli, Sphingomonas paucimobilis, Enterobacter aerogenes,* and *Proteus hauseri.* A study carried out on clinical isolates from patients at the intensive care unit (ICU) of the Burdenko Neurosurgery Institute, Moscow revealed similar findings in *Enterobacter aerogenes and Proteus mirabilis species* as well as in *Escherichia coli*.^28,29^

Carbapenem drugs are not entirely metabolised in the human body and residues are excreted in human excreta, into the hospital sewage system, and eventually into the environment. Through selective pressure and horizontal gene transfer events, pathogenic bacteria in sewer and other environmental organisms are likely to acquire resistance to carbapenems. This may be the reason for the detection of the blaOXA-48 gene in *Sphingomonas Paucimobilis,* an environmental organism commonly isolated from hospital instruments and devices and associated with nosocomial infection, bone and soft tissue infections.^28,30,31^

New Delhi Metallo beta-lactamases (NDM) originally from India, have been disseminated worldwide. In this study, the blaNDM gene was detected from *Escherichia Coli* and *Enterobacter Cloacae*. Similar findings have been documented in a study done during an outbreak at Civil de Guadalajara Fray Antonio Alcalde hospital in Jalisco, Mexico.^26,32^ Klebsiella Pneumoniae Carbapenemase gene (blaKPC) although the most predominant and widely disseminated gene, was not detected from isolates analysed in this study. This is due to the limitations of the study, targeting only bacterial isolates implicated in the causation of diarrheal disease and the fact that very few Klebsiella Pneumoniae isolates were recovered from mobile phones.^32,33^

BlaIMP gene predominantly detected from *Pseudomonas aeruginosa* isolates worldwide and endemic in Japan was not detected. This is in contrast to a study done at Mulago national referral hospital where the gene was detected in archived patients isolates.^23,33^

Identification of organisms implicated in the causation of nosocomial infections, implies that mobile phone use within the hospital is contributing to the spread of nosocomial and multi-drug resistant bacterial strains.^34^

### Study Limitations

Hospitals are considered as unique ecological niches with different microbial biodiversity and varying levels of implementation of infection prevention and control measures, the study outcome therefore cannot be generalized to all hospitals as well as public entities or facilities.

## CONCLUSION

The most common pathogen circulating on the mobile phones of healthcare workers and patients' caretakers at Kitale County hospital is *Enterococcus faecalis*. The detection of the blaOXA-48 gene in *Sphingomonas Paucimobilis* and *Escherichia coli* mobile phone isolates provides proof that mobile phones use in the hospital could be contributing silently to the spread of bacteria with carbapenem resistance genetic determinants.
